# Marked Inter-Unit Variability and Early Depletion of 2,3-Diphosphoglycerate During Red Blood Cell Storage

**DOI:** 10.3390/cells15212028

**Published:** 2026-11-06

**Authors:** Marta Peris, Juan Sahuquillo, Ana Ortuño, Verónica Pons, Marina Rierola, Maria A. Poca

**Affiliations:** 1 Neurotraumatology and Neurosurgery Research Unit, Vall d’Hebron Institut de Recerca (VHIR), Vall d’Hebron Hospital Universitari, Vall d’Hebron Barcelona Hospital Campus, Passeig Vall d’Hebron 119-129, 08035 Barcelona, Spain; pocama@neurotrauma.net; 2 Independent Researcher, 08035 Barcelona, Spain; 3 Department of Hematology, Vall d’Hebron Hospital Universitari, Vall d’Hebron Barcelona Hospital Campus, Passeig Vall d’Hebron 119-129, 08035 Barcelona, Spain; ana.ortuno.cabrero@gmail.com; 4 Blood and Tissue Bank, Vall d’Hebron Hospital Universitari, Vall d’Hebron Barcelona Hospital Campus, Passeig Vall d’Hebron 119-129, 08035 Barcelona, Spain; vpons@bst.cat (V.P.); mrierola@bst.cat (M.R.); 5 Department of Surgery, Vall d’Hebron Barcelona Hospital Campus, Universitat Autònoma de Barcelona, 08035 Barcelona, Spain; 6 Department of Neurosurgery, Vall d’Hebron University Hospital, Passeig Vall d’Hebron 119-129, 08035 Barcelona, Spain

**Keywords:** erythrocyte storage lesion, 2,3-diphosphoglycerate, inter-unit variability, donor variability, liquid chromatography–mass spectrometry, red blood cell quality, blood transfusion

## Abstract

Red blood cell (RBC) transfusion aims to restore tissue oxygen delivery, yet a stored unit’s capacity to unload oxygen depends on 2,3-diphosphoglycerate (2,3-DPG), the principal allosteric modulator of hemoglobin–oxygen affinity. Randomized trials comparing fresher with older units have been consistently null, and all have used storage duration as the exposure variable. We asked whether storage age reflects the metabolic state of individual units. Twenty-four leukodepleted packed RBC units were sampled serially from day 3 to day 42 of standard cold storage. 2,3-DPG was quantified by liquid chromatography–mass spectrometry (LC–MS) with isotopically labelled internal standards, pH was measured in parallel, and trajectories were modelled by linear mixed effects with unit-level random intercepts. At day 3, 2,3-DPG spanned an approximately 18-fold range across units of identical age (0.8–14.7 µmol/g Hb; median 4.0): some were already near-depleted at the earliest sampled timepoint, while others retained near-physiological concentrations. This heterogeneity collapsed rapidly—median values fell to 20% of day-3 levels by day 7 and by more than 95% by day 14 (β = −0.298 µmol/g Hb per day, *p* < 0.001)—and were below the limit of detection in most units after day 17. pH declined monotonically to 6.40 ± 0.04 by day 42 (β = −0.0163 units per day, *p* < 0.001). Units of identical storage age therefore differ markedly in the levels of the metabolite that governs oxygen unloading, and are depleted long before the 42-day expiry limit, suggesting that storage duration alone may incompletely capture the metabolic state of stored red blood cell units.

## 1. Introduction

The primary objective of blood transfusion is to maintain or restore adequate oxygen delivery to tissues. However, prolonged storage under blood bank conditions leads to the development of the so-called “storage lesion”, a multifactorial process characterized by progressive biochemical, metabolic, and structural deterioration of red blood cells (RBCs). These changes include progressive metabolic depletion, disturbances in acid–base homeostasis, and increasing cytoskeletal instability [1,2]. During storage, the continued reliance of RBCs on glycolysis leads to gradual acidification of the suspension medium, a process widely recognized as a central mediator of storage associated metabolic derangement and membrane remodeling [1,3,4]. This acidic milieu compromises cytoskeletal integrity, resulting in increased osmotic fragility (OF) and a higher rate of time-dependent hemolysis [1,3]. Consistent with this, Peris et al. recently demonstrated that OF increases significantly after 28 days of storage when RBCs are exposed to hypotonic stress, reflecting progressive impairment of membrane stability [3].

2,3-Diphosphoglycerate (2,3-DPG) is produced via the Rapoport–Luebering shunt, a glycolytic side pathway largely restricted to mammalian RBCs. As a potent allosteric modulator of hemoglobin (Hb), 2,3-DPG binds reversibly to deoxygenated Hb, reduces its oxygen affinity, and thereby facilitates oxygen release to peripheral tissues [5,6,7]. Under standard blood bank storage conditions, 2,3-DPG concentrations decline progressively, reaching very low levels after approximately two weeks and becoming nearly depleted by 21 days of storage [7,8]. The loss of 2,3-DPG increases hemoglobin–oxygen affinity, resulting in a leftward shift in the oxyhemoglobin dissociation curve and a reduction in P_50_, the partial pressure of O_2_ at which hemoglobin is 50% saturated. Consequently, oxygen unloading to peripheral tissues may be impaired. Because of this physiological role, 2,3-DPG depletion has traditionally been considered a key metabolic component of the RBC storage lesion and a potential determinant of oxygen unloading capacity following transfusion. According to the Siggaard-Andersen classification, increased hemoglobin–oxygen affinity (reduced P_50_) may contribute to a form of tissue hypoxia termed “high-affinity hypoxia”, in which arterial oxygen tension and hemoglobin concentration are normal, but oxygen unloading and tissue extraction are impaired despite adequate arterial oxygen content [9,10].

Metabolomic studies have characterized the metabolic remodeling of stored RBCs in considerable detail [4,11], and have demonstrated substantial donor-dependent heterogeneity in the development of the storage lesion [12,13]. However, the degree of variability in 2,3-DPG concentrations among RBC units of identical storage age has not been systematically quantified using longitudinal measurements and absolute quantification methods. To address this gap, we applied targeted liquid chromatography–mass spectrometry (LC-MS) with isotopically labelled internal standards, which enables accurate quantification of phosphorylated glycolytic intermediates at the low concentrations encountered during prolonged storage [14,15,16], to serially sampled leukodepleted RBC units. Our objectives were to quantify the magnitude of inter-unit variability in 2,3-DPG at a fixed storage age, describe the temporal trajectory of its depletion, and examine its relationship with storage-associated acidification. Because 2,3-DPG is a major determinant of hemoglobin–oxygen affinity, the extent to which units of the same nominal storage age differ in this metabolite raises the possibility that storage duration alone may not adequately reflect the metabolic quality of transfused RBC units and may therefore influence the interpretation of studies evaluating the effects of RBC storage duration.

## 2. Materials and Methods

This work is part of a broader research program conducted by the Neurosurgery and Neurotraumatology Research Unit at Vall d’Hebron University Hospital, dedicated to advancing strategies that optimize oxygen transport in neurocritical care. Ethical approval for the full project was granted by the Ethics Committee of the Vall d’Hebron Research Institute (protocol ID: PR AG 411/2017, approval date: 6 June 2017). All procedures were carried out in accordance with Spanish law and the relevant European Union directives.

### 2.1. Leukodepleted Packed Red Blood Cell Concentrates (pRBCs)

Twenty-four leukodepleted pRBC units derived from 24 different healthy volunteer donors were obtained from our institution’s Blood and Tissue Bank in accordance with all applicable regulatory requirements, including EU Regulation 2016/679 governing personal data protection. Approximately 450 mL of whole blood was collected in CPD anticoagulant. Following centrifugation, leukocytes and plasma were removed, and the resulting RBC fraction was resuspended in 100 mL of SAGM (Saline, Adenine, Glucose, and Mannitol) storage solution. The pRBC units were delivered to the research team 3 days after donation, maintained at 1–6 °C, and sampled from storage day 3 through day 42. Day 3 represented the earliest time point available for analysis and should not be considered a biological baseline, as changes occurring during the first 72 h after donation were not assessed. Donor demographic and hematological characteristics (e.g., age, sex, ABO blood group, and hemoglobin concentration) were not available to the investigators because all units were supplied in anonymized form by the Blood and Tissue Bank, precluding assessment of their contribution to inter-unit variability.

### 2.2. Liquid Chromatography–Mass Spectrometry

At each sampling time point (Days 3, 7, 10, 14, 17, 21, 28, 35, and 42), a 250 μL aliquot of pRBC concentrate was collected from the sampling port of the blood bag into a heparinized collection tube to prevent coagulation. Each sample was immediately mixed with 500 μL of 1× phosphate-buffered saline (PBS; pH 7.4) and 30 μL of perchloric acid (HClO_4_). Centrifugation at 12,000 rpm for 5 min at 4 °C produced a brown pellet and a clear, slightly light brown supernatant. A 500 μL aliquot of the supernatant was then neutralized with 56 μL of 2.5 M potassium carbonate (K_2_CO_3_), generating a characteristic white precipitate. The mixture was then centrifuged again at 4 °C and 5000 rpm for 10 min, and 400 μL of the final supernatant was collected and stored at −80 °C, the optimal temperature for metabolite preservation [16]. Preprocessed samples were maintained on dry ice during transport to the MarMetabolomics facility at the Hospital del Mar Research Institute Barcelona (HMRIB), where the analyses were performed. Upon arrival, 20 µL of sample was mixed with 10 µL of internal standard consisting of ADP-^13^C5 (10 µg/mL), 120 µL of water, and 350 µL of acetonitrile.

2,3-DPG was determined by LC-MS/MS using a Waters ACQUITY UPLC system (Waters Corporation, Milford, MA, USA) coupled to a Waters Xevo TQS Micro triple-quadrupole mass spectrometer (Waters Corporation, Milford, MA, USA) equipped with an electrospray ionization source. Separation was performed using an ACQUITY UPLC BEH Amide column (1.7 µm, 2.1 × 100 mm; Waters Associates) at a flow rate of 600 µL/min. Quantification of 2,3-DPG was performed by external calibration using the transitions 265 → 167 and 265 → 79 in negative ionization mode, with the analyte-to-internal-standard peak-area ratio used as the analytical response. The limit of quantification (LOQ) of the method was 0.05 µmol/mL, and the limit of detection (LOD) was 0.02 µmol/mL. MassLynx software version 4.1 and TargetLynx XS version 4.1 were used for data management.

2,3-DPG concentrations were expressed relative to hemoglobin (μmol/g Hb), as this normalization reflects the physiologically relevant interaction between 2,3-DPG and hemoglobin while minimizing variability related to differences in hematocrit and red cell mass [17]. This approach facilitates direct comparison of the metabolic status of individual samples independent of cell count. Total hemoglobin (tHb) concentration was determined using a GEM^®^ Premier™ 4000 blood gas analyzer (Instrumentation Laboratory, Werfen, Bedford, MA, USA). For this purpose, an additional 1.5 mL blood sample was collected into a heparinized syringe and analyzed using the instrument.

### 2.3. Potential of Hydrogen (pH)

The measurement of pH was carried out using a specialized blood sample pH meter (InLab Micro Pro-ISM, Mettler-Toledo, Cornellà de Llobregat, Spain). The blood units were stored under the same conditions as the blood bank, at 4 °C. However, pH measurements were taken at room temperature (20–23 °C) to determine the pH of the bags under the conditions of routine blood transfusions. Ensuring that the measurement temperature was very similar between measurements was considered essential, as it is a factor that influences the final pH value. Maintaining a consistent measurement temperature was considered essential because pH is temperature-dependent. Importantly, the measured values represent the extracellular pH of the storage medium and should not be interpreted as direct measurements of intracellular erythrocyte pH. In the context of this study, extracellular pH was used as an indicator of storage-associated acidification.

### 2.4. Statistical Analysis

All statistical analyses were performed using R (version 4.5.2) [18] in the RStudio integrated development environment (version 2026.01.2) [19]. Descriptive statistics were calculated for all variables. Distributional properties were evaluated using exploratory graphical methods, including histograms and Q–Q plots, with particular emphasis on the distribution of model residuals. Variables with approximately symmetric distributions are presented as mean ± standard deviation (SD), whereas skewed variables are summarized as median and range. Individual 2,3-DPG trajectories were visualized using spaghetti plots to characterize within-unit changes during storage, and boxplots were used to summarize the distributions of 2,3-DPG and pH at each storage time point. All figures were generated in R using the ggplot2 package [20].

**Linear Mixed-Effects Modeling.** Because 2,3-DPG and pH were measured repeatedly within the same pRBC units, longitudinal changes were analyzed using linear mixed-effects models (LMMs). Random intercepts for individual pRBC units were included to account for within-unit correlation arising from repeated measurements and to accommodate the unbalanced number of observations across units. Two LMMs were fitted:

- 2,3-DPG model: 2,3-DPG ~ storage day + (1 | pRBC unit), to evaluate the association between storage duration and 2,3-DPG concentration.
- pH model: pH ~ storage day + (1 | pRBC unit), to evaluate the association between storage duration and pH.

Model assumptions, including linearity, normality of residuals, and homoscedasticity, were evaluated using diagnostic plots.

**Fixed-Effect Testing and Inference.** Models were fitted using the *lme4* package [21]. Fixed effects were assessed using Wald t-tests with Satterthwaite approximations for degrees of freedom, as implemented in the *lmerTest* package [22]. Statistical significance was defined as *p* < 0.05. Model-based estimated marginal means and contrasts were obtained using the emmeans package when appropriate [23]. For comparisons of individual storage time points with Day 3, storage day was treated as a categorical factor, and confidence intervals and *p* values were adjusted for multiple comparisons using Dunnett’s method.

**Data Management.** The study adhered to FAIR data principles [24]. The complete anonymized dataset, metadata, variable dictionary, and R code used for all analyses are publicly available through Zenodo (DOI: 10.5281/zenodo.20786075) and the associated GitHub repository (accessed on 5 September 2026).

## 3. Results

### 3.1. 2,3-DPG Dynamics During Storage

**Figure 1:**
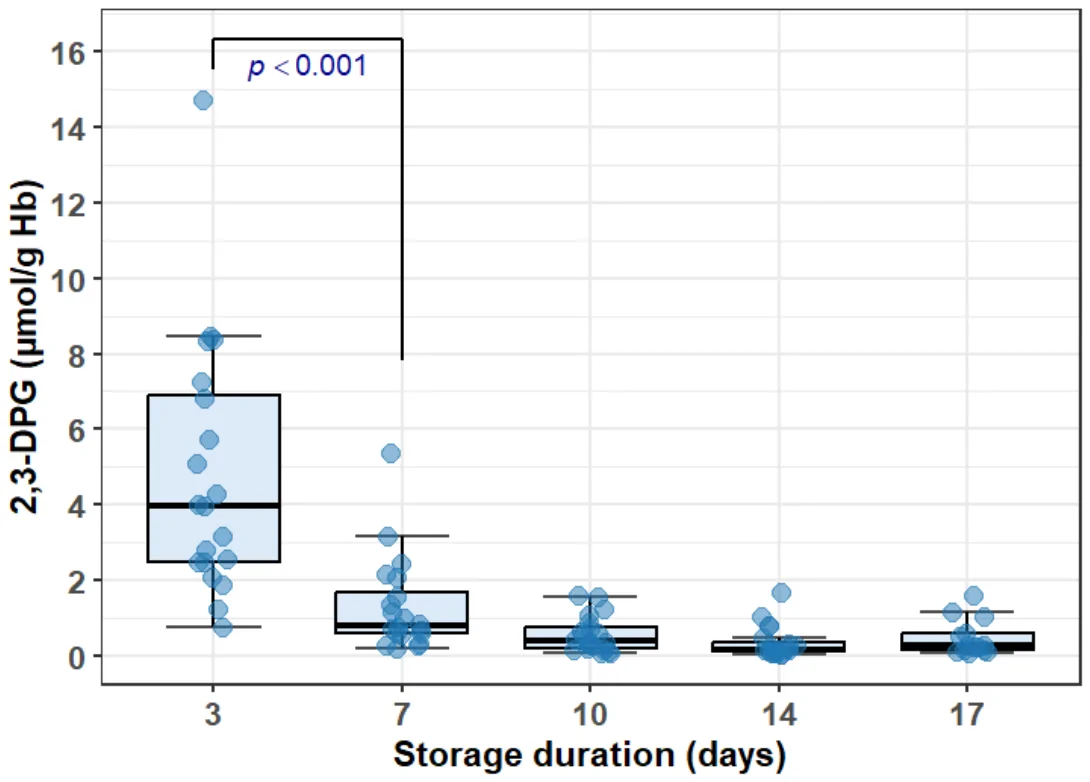
Changes in 2,3-DPG concentrations during RBC storage. Boxplots and individual data points show 2,3-DPG concentrations (µmol/g Hb) measured from Day 3 through Day 17 of storage. Marked inter-unit variability was observed at the earliest available sampling time point (Day 3), followed by a progressive decline in 2,3-DPG concentrations during storage. After Day 17, most 2,3-DPG concentrations fell below the limit of quantification (LOQ = 0.05 µmol/mL) of the assay and are therefore not shown. The asterisk denotes the model-based contrast between Days 3 and 7 (*p* < 0.001). Figures are limited to Day 17 because most 2,3-DPG concentrations fell below the assay LOQ thereafter.

**Figure 2:**
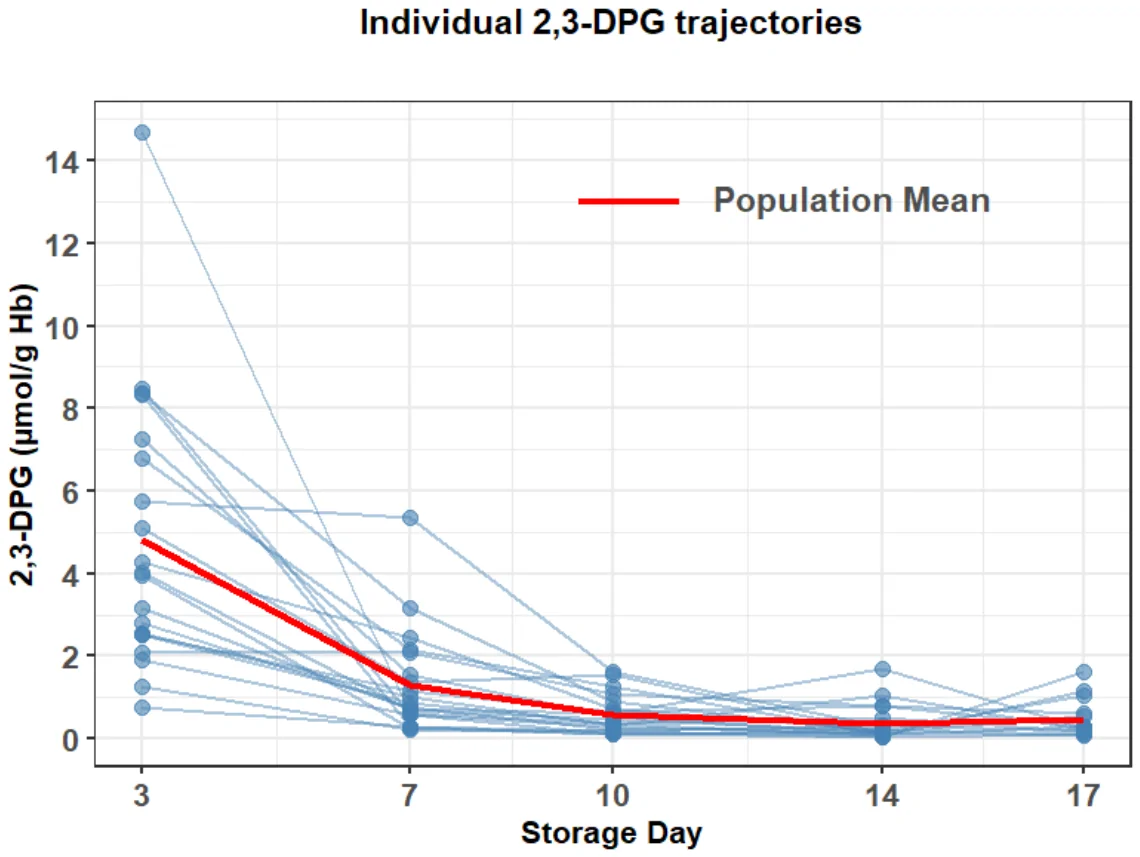
Spaghetti plot displaying individual 2,3-DPG trajectories (thin blue lines) for each leukodepleted pRBC unit. Although 2,3-DPG concentrations varied substantially among units at the earliest sampling time point (Day 3), most units exhibited an early decline during storage, with considerable variability in the rate and magnitude of depletion. The steepest reductions generally occurred between Days 3 and 7. The population-level mean trajectory (thick red line) illustrates the overall progressive decline in 2,3-DPG concentrations over time, culminating in near-complete depletion by Days 14–17. The figure also highlights the marked inter-unit variability observed at Day 3 despite the common tendency toward progressive depletion during subsequent storage.

2,3-DPG concentrations declined rapidly during storage across leukodepleted pRBC units. At the earliest available sampling time point (Day 3), the median 2,3-DPG concentration was 3.99 µmol/g Hb (range, 0.77–14.7 µmol/g Hb), indicating substantial inter-unit variability early in the storage period. Despite this initial dispersion, most units exhibited a pronounced early decline in 2,3-DPG concentrations, although the magnitude and timing of the decline varied among units. By Day 7, the median concentration had decreased to 0.81 µmol/g Hb (range, 0.22–5.37 µmol/g Hb), corresponding to 20.3% of the Day 3 median value (Figure 1 and Figure 2). Concentrations continued to decrease thereafter, with median values of 0.42 µmol/g Hb at Day 10 and 0.18 µmol/g Hb at Day 14, representing a 95.5% reduction from the Day 3 median. At Day 17, the median concentration was 0.27 µmol/g Hb among the 14 samples with quantifiable measurements. After Day 17, most samples yielded 2,3-DPG concentrations below the limit of quantification (LOQ = 0.05 µmol/mL). Spaghetti plots demonstrated marked heterogeneity at Day 3 but a predominantly downward trajectory thereafter, with the steepest decline occurring between Days 3 and 7 (Figure 2).

**Linear Mixed-Effects Modeling.** A linear mixed-effects model with random intercepts demonstrated a strong association between storage duration and 2,3-DPG concentration. Storage day was significantly associated with lower 2,3-DPG concentrations (β = −0.298 µmol/g Hb per day, robust SE = 0.053; t(18.1) = −5.59; *p* < 0.001), corresponding to an estimated weekly decrease of approximately 2.1 µmol/g Hb. Model-based contrasts estimated a decrease of 1.19 µmol/g Hb between Days 3 and 7 (95% CI, 0.75–1.64; *p* < 0.001) and 4.17 µmol/g Hb between Days 3 and 17. Together with the descriptive analyses, these findings indicate a marked decline in 2,3-DPG concentrations during storage, with the most rapid depletion occurring during the first week and near-complete depletion by Days 14–17.

### 3.2. Evolution of pH During Storage

**Figure 3:**
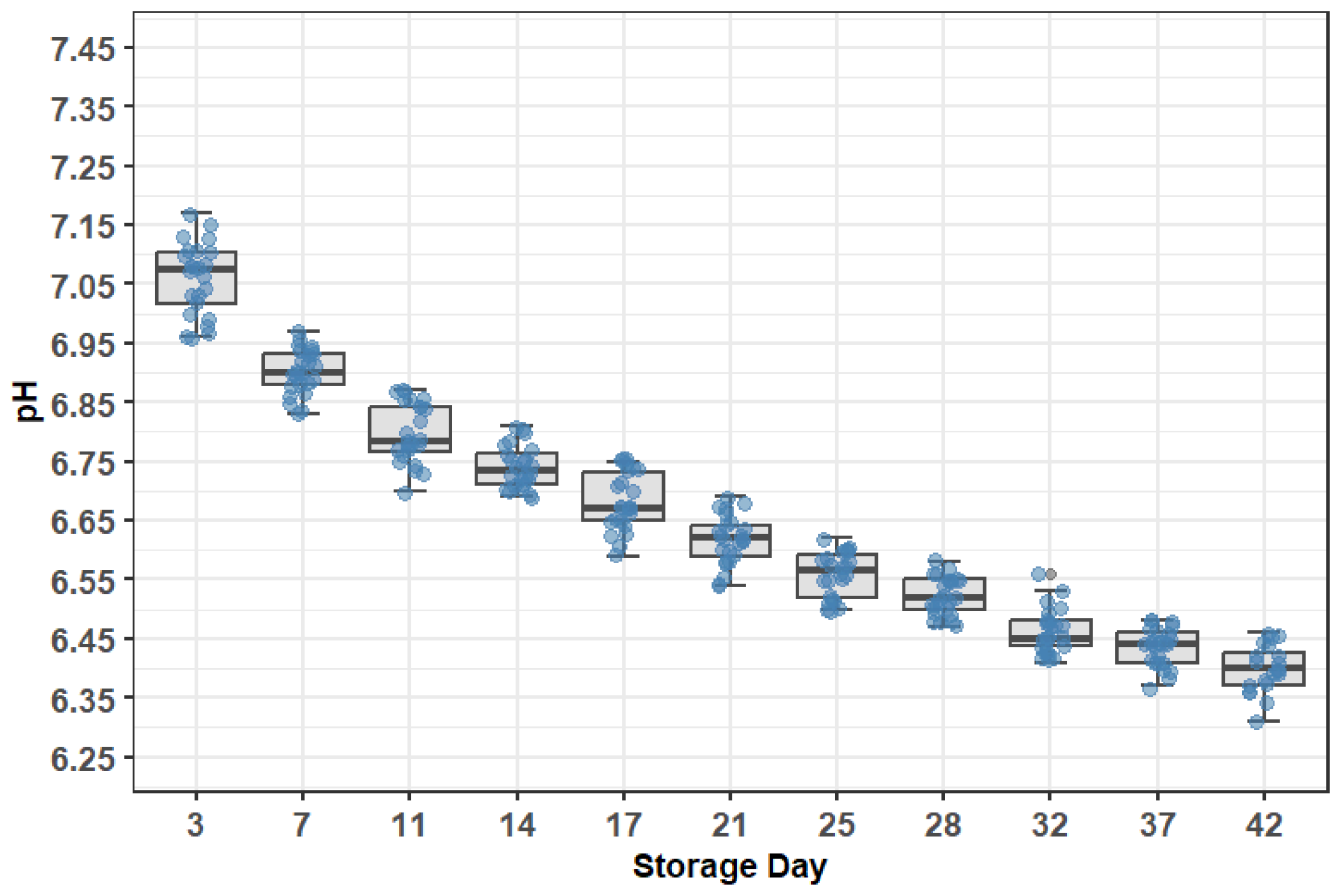
Trajectory of pH values during storage of leukodepleted red blood cell units. Individual measurements (blue points) and corresponding boxplots demonstrate a progressive decline in pH from Day 3 to Day 42. A linear mixed-effects model confirmed a strong association between storage duration and pH (β = −0.0163 pH units/day; *p* < 0.001), consistent with progressive storage-associated acidification (Table 1).

The dataset included pH measurements from 24 pRBC units across 11 storage days (Days 3–42). On Day 3, the mean pH was 7.06 ± 0.06 (range, 6.96–7.17). A progressive and nearly monotonic decline was observed thereafter, with mean pH reaching 6.40 ± 0.04 (range, 6.31–6.46) by Day 42. Boxplots with individual data points (Figure 3) showed limited inter-unit variability throughout storage, with a consistent shift toward lower pH values over time.

A linear mixed-effects model fitted to 260 observations from 24 pRBC units demonstrated a strong association between storage duration and declining pH. Increasing storage time was associated with a mean decrease of 0.0163 pH units per day (β = −0.0163; SE = 0.00033; *p* < 0.001), corresponding to an average decline of approximately 0.11 pH units per week.

To quantify the magnitude of acidification at clinically relevant time points, model-based contrasts comparing selected storage days with Day 3 were calculated (Table 1). pH was significantly lower than at Day 3 at all subsequent time points. By Day 7, pH had decreased by Δ = −0.157 (95% CI, −0.184 to −0.130; *p* < 0.0001). The difference from Day 3 increased progressively, reaching Δ = −0.320 by Day 14 (95% CI, −0.347 to −0.293; *p* < 0.0001) and Δ = −0.443 by Day 21 (95% CI, −0.470 to −0.416; *p* < 0.0001). By Day 42, pH was 0.655 units lower than at Day 3 (Δ = −0.655; 95% CI, −0.684 to −0.627; *p* < 0.0001), consistent with progressive storage-related acidification. Overall, the magnitude of the difference from Day 3 increased monotonically with storage duration.

## 4. Discussion

At the tissue level, O_2_ release from Hb is governed by the Hb–O_2_ dissociation curve, which is strongly modulated by the erythrocyte metabolite 2,3-DPG. The allosteric role of 2,3-DPG in modulating Hb–O_2_ affinity was first established by Benesch and Benesch in 1967 [6]. Subsequent work, including that of MacDonald, further expanded these findings by integrating physiological and clinical perspectives [25]. By binding preferentially to deoxygenated hemoglobin, 2,3-DPG stabilizes the low-affinity tense state, thereby facilitating oxygen unloading to peripheral tissues. Using LC–MS, a reference analytical method, we showed that stored RBCs undergo a rapid, early, and time-dependent decline in 2,3-DPG levels. Notably, this early phase was characterized by substantial inter-unit variability in baseline 2,3-DPG concentrations, detectable as early as Day 3 of storage, before a uniform downward trajectory emerged across all units. The decline was most pronounced during the first week post collection, culminating in near-complete depletion within two weeks of storage. These results confirm and extend previous reports of progressive 2,3-DPG depletion during RBC storage, with higher resolution temporal and quantitative characterization than enzymatic assay-based studies [7,8,14,25,26,27,28].

The marked inter-unit variability in 2,3-DPG concentrations observed at the earliest available sampling time point (Day 3 post-donation) was one of the most striking findings of the study and challenges the common assumption that storage duration alone adequately reflects the metabolic quality of stored RBC units. Although all pRBC units were of identical storage age, 2,3-DPG concentrations varied approximately 18-fold at Day 3, indicating substantial metabolic heterogeneity early in the storage period (Figure 1 and Figure 2). The severity and progression of the RBC storage lesion are known to vary considerably among units and may be influenced by several factors, including blood-processing procedures, storage additive solutions, and donor-related characteristics such as age, sex, genetic background, and donation history [12,13,29]. We hypothesize that the marked variability observed at Day 3 may reflect differences in donor biology, erythrocyte age distribution, glycolytic activity, or processing-related factors occurring before the first measurement. Because the present study did not collect donor-specific or manufacturing data, the relative contribution of these factors could not be determined and warrants further investigation. A better understanding of donor-related variability in RBC metabolism and subpopulation composition may facilitate the optimization of pRBC manufacturing processes and improve the characterization of unit quality beyond storage duration alone. Future studies should investigate donor-related determinants of baseline 2,3-DPG concentrations and characterize changes occurring during the first 72 h after donation, a critical period that was not assessed in the present study.

The mechanisms underlying 2,3-DPG depletion during RBC storage are well established. Stored RBCs rely exclusively on anaerobic glycolysis for energy production and therefore continuously generate lactate within a closed system, without access to the Cori cycle for lactate recycling. Progressive lactate accumulation during storage promotes acidification of the storage environment and is expected to be accompanied by intracellular acidification, which has been shown to disrupt glycolytic regulation and promote 2,3-DPG degradation [4,11,30]. In the present study, pH was measured in the extracellular storage medium and therefore provides an indirect indicator of these metabolic changes. Consistent with these mechanisms, we observed a monotonic decline in storage pH, with mean values reaching 6.40 ± 0.04 by Day 42. Previous experimental studies have suggested that storage-associated acidification may contribute to 2,3-DPG depletion by inhibiting 2,3-DPG mutase and promoting the activity of 2,3-DPG phosphatase [30,31]. Although this mechanism is biologically plausible and supported by prior literature, the present study did not directly assess intracellular pH, glycolytic intermediates, or enzyme activity. Consequently, our findings should be interpreted as being consistent with, rather than demonstrating, these proposed mechanisms.

Based on storage duration, RBC units are frequently labeled as “young” (<14–21 days of storage) or “old” (>21 days of storage); however, these designations are largely arbitrary, lack standardized biological justification, and are inconsistently applied across studies, thereby limiting their interpretability and comparability [32,33]. In a meta-analysis including 21 studies—only three of which were randomized—Wang et al. reported that transfusion of older RBC units was associated with a significantly increased risk of mortality (odds ratio 1.16; 95% confidence interval 1.07–1.24) [34]. In contrast, a subsequent meta-analysis by Cheng et al., encompassing 26 randomized controlled trials with low risk of bias, found that transfusion of fresher RBCs did not reduce mortality, transfusion-related adverse events, or length of hospital stay across multiple clinical settings, including critical care and cardiac surgery [33]. Despite the absence of a definitive clinical answer to this question, it is evident that under standard cold storage conditions, the quality of stored blood deteriorates progressively over time. Reduced 2,3-DPG levels represent only one of several biochemical signatures of RBCs stored for longer than two weeks. This depletion is expected to increase hemoglobin–oxygen affinity, although the functional consequences were not evaluated in the present study. Although 2,3-DPG depletion is expected to increase hemoglobin–oxygen affinity, the net physiological consequences of this effect following transfusion remain uncertain. Factors such as post-transfusion regeneration of 2,3-DPG, the proportion of transfused RBCs relative to the total circulating erythrocyte mass, and concurrent acid–base effects may all influence oxygen unloading capacity. Therefore, the clinical significance of isolated 2,3-DPG depletion in stored pRBCs remains uncertain and should be evaluated in studies incorporating direct measurements of hemoglobin–oxygen affinity, oxygen unloading, tissue oxygenation, and clinical outcomes. Consequently, the apparent lack of clinical benefit of transfusing fresher versus older blood should be interpreted with caution, as RBCs stored for more than two weeks already exhibit substantial biochemical deterioration, and further storage beyond approximately 28 days is associated with increased osmotic fragility and progressive membrane instability [3].

Several experimental approaches have been investigated to preserve intracellular 2,3-DPG levels during RBC storage. As early as the 1970s, Wood and Beutler demonstrated that supplementation of storage solutions with sodium ascorbate could substantially preserve intracellular 2,3-DPG concentrations during prolonged refrigerated storage [28]. Subsequent studies have shown that stabilizing storage pH, most notably through removal of CO_2_ using an internal absorbent trap, can partially attenuate the decline in intracellular 2,3-DPG levels during pRBC storage [26]. In parallel, biochemical rejuvenation and oxygen-controlled storage strategies have been explored as means of preserving ATP and 2,3-DPG during prolonged storage. Rejuvenation with metabolic substrates such as pyruvate, inosine, adenine, and phosphate can restore intracellular energy stores late in storage, whereas anaerobic or hypoxic storage may limit oxidative and acidotic stress, thereby helping to preserve glycolytic function and 2,3-DPG concentrations [8,14,26,27,28,32,35,36]. Collectively, these studies suggest that storage-associated depletion of 2,3-DPG may represent a modifiable component of the RBC storage lesion. However, whether these interventions can reliably preserve RBC metabolic quality, improve oxygen delivery, or translate into clinically meaningful benefits remains uncertain and warrants further investigation.

## 5. Study Limitations

Several limitations of this study should be acknowledged. First, the study was conducted at a single institution and included a relatively small number of pRBC units (*n* = 24). Second, all units were processed using the same manufacturing procedures and stored in SAGM additive solution, which may limit the generalizability of the findings to other blood-processing protocols and storage systems. Third, the first measurement was performed on Day 3 after donation; therefore, changes occurring during the first 72 h of storage, a period during which substantial 2,3-DPG depletion may occur, could not be assessed. Fourth, most 2,3-DPG concentrations fell below the limit of quantification after Day 17, precluding reliable characterization of inter-unit variability during the later stages of storage. Finally, the study did not include measurements of P50, hemoglobin–oxygen affinity, oxygen unloading, tissue oxygenation, lactate, ATP, intracellular pH, or clinical outcomes. Consequently, the physiological and clinical significance of the observed metabolic heterogeneity remains to be established.

## 6. Conclusions

Using LC-MS as a reference analytical method, we provide a high-resolution characterization of the timing and extent of 2,3-DPG depletion during RBC storage. Our results demonstrate that substantial metabolic alterations arise early during storage and that marked inter-unit variability exists among pRBC units of identical storage age. Notably, 2,3-DPG concentrations varied approximately 18-fold among units at the earliest available sampling time point, indicating that RBC units of identical chronological age may differ substantially in their metabolic state. Together, these findings suggest that storage duration alone may not adequately reflect the metabolic quality of individual pRBC units.

Although the observed decline in 2,3-DPG occurred in parallel with progressive storage-associated acidification, the present study was not designed to establish the mechanistic basis of this relationship. Furthermore, the marked variability in 2,3-DPG concentrations observed among units of identical storage age suggests that donor-related and processing-related factors may contribute substantially to the metabolic heterogeneity of stored RBCs. Further studies incorporating direct measurements of intracellular metabolism, hemoglobin–oxygen affinity, oxygen unloading, tissue oxygenation, and clinical outcomes are required to determine the physiological and clinical significance of these findings. Our findings challenge the implicit assumption that storage duration is an adequate surrogate for the metabolic state of stored RBC units and support the need for biologically informed approaches to the assessment of RBC storage quality.

Finally, LC-MS-based metabolomic profiling provides a robust framework for future investigations of RBC storage biology and may facilitate the integration of biochemical, structural, functional, and clinical endpoints in the evaluation of strategies aimed at preserving RBC quality during storage.

## 7. Transparency, Rigor and Reproducibility Summary

**Study registration**. This study was not formally pre-registered. It was conducted as part of a broader ethically approved research program (protocol ID: PR AG 411/2017, Ethics Committee of the Vall d’Hebron Research Institute), and the primary objective and analytical approach were defined prior to data collection.

**Analytic plan specification**. The analysis plan was not formally pre-registered. However, the team member with primary responsibility for statistical analysis (J. Sahuquillo) certifies that the plan—including the use of linear mixed-effects models (LMMs) with random intercepts for pRBC unit, separate models for storage day and pH as predictors, and Satterthwaite degrees of freedom for fixed-effect inference—was pre-specified before analysis commenced.

**Statistical power and sample size**. A sample of 24 leukodepleted pRBC units was planned based on availability of units from the Blood and Tissue Bank and on logistical constraints inherent to the storage protocol. Formal a priori power calculations were not performed, as no published effect size estimates for LC–MS-based 2,3-DPG quantification in SAGM-stored pRBC units were available at the time of study design. The observed effect size for the storage day predictor was β = −0.298 µmol/g Hb per day (robust SE = 0.053; t(18.1) = −5.59; *p* < 0.001).

**Subject accounting.** Twenty-four pRBC units were obtained and included in the study. All 24 units were available for 2,3-DPG analyses throughout the study period, and no units were excluded from these analyses. After Day 17, 2,3-DPG concentrations fell below the lower limit of quantification (LLOQ) in most units; these observations were excluded from LMM fitting for that analyte. pH measurements were obtained from all 24 units throughout most of the storage period; however, four units were unavailable for pH assessment at Day 42 due to COVID-19-related disruptions.

**Randomization**. Randomization was not applicable, as pRBC units were not assigned to intervention groups. Units were processed and sampled sequentially according to the storage timeline.

**Blinding**. LC–MS analyses were performed at an external laboratory (Applied Metabolomics Laboratory, IMIM) by investigators not involved in study design or data interpretation, providing de facto analytical blinding. pH measurements were performed without knowledge of LC–MS results at the time of measurement.

**Experimental conditions**. pRBC units were maintained at 1–6 °C throughout storage. Samples for LC–MS were collected using heparinized tubes, preprocessed immediately, and stored at −80 °C prior to batched analysis. pH measurements were performed at room temperature (20–23 °C) to reflect conditions at the time of transfusion. All LC–MS analyses were performed in a single batch at IMIM using an ACQUITY UPLC/Xevo TQS Micro system with isotopically labelled internal standards.

**Analytical characteristics**. Quantification was performed by external calibration with defined analyte concentrations, complemented by ^13C-substituted internal standards added to all calibration solutions and sample matrices. The limit of quantification (LOQ) of the method was 0.05 µmol/mL, and the limit of detection (LOD) was 0.02 µmol/mL. Samples with concentrations below the LOQ after Day 17 were excluded from LMM analyses.

**Replication**. No external replication or validation studies have been performed or are planned at this time.

**Data and code availability**. The complete anonymized dataset, metadata, variable dictionary, and R code used for all statistical analyses are available at https://doi.org/10.5281/zenodo.20786075 and at the corresponding GitHub repository: https://github.com/Sahuquillo/dpg-prbc-storage-lesion (accessed on 5 September 2026). Both repositories are linked and cross-referenced.

## Figures and Tables

**Table 1:** Model-based contrasts in pH at selected storage days relative to Day 3. Linear mixed-effects model with bag-level random intercepts. Estimated marginal means (EMMs) with 95% confidence intervals (CIs). Negative values indicate lower pH compared with Day 3.

Storage Day Comparison	Δ pH (Estimate)	95% CI	SE	df	*p*-Value
**DAY 7 vs. DAY 3**	−0.157	−0.184 to −0.130	0.00981	226	<0.0001
**DAY 14 vs. DAY 3**	−0.320	−0.347 to −0.293	0.00981	226	<0.0001
**DAY 21 vs. DAY 3**	−0.443	−0.470 to −0.416	0.00981	226	<0.0001
**DAY 42 vs. DAY 3**	−0.655	−0.684 to −0.627	0.01030	227	<0.0001

## Data Availability

The complete anonymized dataset, metadata, variable dictionary, and R code used for all statistical analyses are available at https://doi.org/10.5281/zenodo.20786075 and at the corresponding GitHub repository: https://github.com/Sahuquillo/dpg-prbc-storage-lesion (accessed on 5 September 2026). Both repositories are linked and cross-referenced.

## Abbreviations

The following abbreviations are used in this manuscript:

2,3-DPG	2,3-diphosphoglycerate
ANOVA	Analysis of variance
CPD	Citrate-phosphate-dextrose
ESI	Electrospray ionization
Hb	Hemoglobin
HClO_4_	Perchloric acid
K_2_CO_3_	Potassium carbonate
LC-MS	Liquid chromatography–mass spectrometry
LMM	Linear mixed-effects modeling
OF	Osmotic fragility
PBS	Phosphate-buffered saline
pH	Potential of hydrogen
pRBC	Packed red blood cells
RBC	Red blood cell
SAGM	Saline, Adenine, Glucose and Mannitol
SD	Standard deviation
